# Long-Term Efficacy of Extracorporeal Shock Wave Therapy on Lower Limb Post-Stroke Spasticity: A Systematic Review and Meta-Analysis of Randomized Controlled Trials

**DOI:** 10.3390/jcm10010086

**Published:** 2020-12-29

**Authors:** Emanuela Elena Mihai, Luminita Dumitru, Ilie Valentin Mihai, Mihai Berteanu

**Affiliations:** 1 Physical and Rehabilitation Medicine Department, Carol Davila University of Medicine and Pharmacy Bucharest, 050451 Bucharest, Romania; luminita.dumitru@umfcd.ro (L.D.); mihai.berteanu@umfcd.ro (M.B.); 2 Physical and Rehabilitation Medicine Department, Elias University Emergency Hospital, 011461 Bucharest, Romania; 3 Doctoral School of Electronics, University Politehnica of Bucharest, 060042 Bucharest, Romania; ilie-valentin.mihai@univ-rennes1.fr; 4 Institute of Electronics and Telecommunications of Rennes, University of Rennes 1, 35000 Rennes, France

**Keywords:** extracorporeal shock wave therapy, neurological rehabilitation, stroke, spasticity, hemiplegia

## Abstract

The purpose of this systematic review and meta-analysis is to evaluate the long-term efficacy of Extracorporeal Shock Wave Therapy (ESWT) on reducing lower limb post-stroke spasticity in adults. A systematic electronic search of PubMed/ MEDLINE, Physiotherapy Evidence Database (PEDro), Scopus, Ovid MEDLINE(R), and search engine of Google Scholar was performed. Publications that ranged from January 2010 to August 2020, published in English, French, Spanish, Portuguese, and Italian language and available as full texts were eligible for inclusion and they were searched without any restrictions of country. The study was conducted according to the Preferred Reporting Items for Systematic Reviews and Meta-analyses (PRISMA) guidelines and followed the recommendations of the Cochrane Handbook for Systematic Reviews of Interventions. Two authors screened the references, extracted data, and assessed the risk of bias. The primary outcome was spasticity grade mainly assessed by the Modified Ashworth Scale (MAS). Secondary outcomes were passive range of motion (PROM), pain intensity, electrophysiological parameters, gait assessment, and adverse events. A total of seven recent randomized controlled trials (RCTs) were included in the systematic review and meta-analysis, and a beneficial effect on spasticity was found. The high level of evidence presented in this paper showed that ESWT ameliorates spasticity considering the parameters: MAS: standardized mean difference (SMD) = 0.53; 95% confidence interval (95% CI): (0.07–0.99); Modified Tardieu Scale (MTS): SMD = 0.56; 95% CI: (0.01–1.12); Visual Analogue Scale (VAS): SMD = 0.35; 95% CI: (−0.21–0.91); PROM: SMD = 0.69; 95% CI: (0.20–1.19). ESWT presented long-term efficacy on lower limb post-stroke spasticity, reduced pain intensity, and increased range of motion. The effect of this novel and non-invasive therapy was significant and the intervention did not present adverse events, proving a satisfactory safety profile.

## 1. Introduction

Over the past decade, death caused by cerebrovascular accidents (stroke) has significantly decreased, but stroke is ranked as the second leading cause of death worldwide with an annual mortality rate of 5.5 million and the third leading cause of disability [1,2,3].

Following stroke, patients may often present diverse sensory-motor disturbances, such as spasticity, muscle weakness, or impaired sensation [4,5,6]. Lance defines spasticity as a motor disorder that is characterized by a velocity-dependent increase in tonic stretch reflexes (muscle tone) with exaggerated tendon jerks due to the hyperexcitability of the stretch reflex, as a component of the upper motor neuron syndrome (UMNS) [7]. The definition highlights the fact that spasticity plays the role of a component of UMNS. In patients with UMNS, hypertonia can be classified, as follows: hypertonia that is mediated by the stretch reflex, which corresponds to spasticity and hypertonia due to soft tissue changes, which corresponds to nonreflex hypertonia or intrinsic hypertonia [8]. Spasticity is more often present in the flexor muscles of the upper limb and in the extensor muscles of the lower limb [8].

Spasticity affects 20% to 40% of stroke survivors and the economical and physical burden are substantial, having a great impact not only on patients, but also on caregivers and society [9]. Stroke survivors presenting spasticity may experience pain, impaired motor function, and reduced range of motion (ROM). Therefore, these aspects can lead to reductions in their ability to perform activities of daily living (ADL) and substantially reduce patient health-related quality of life [10,11,12,13,14].

Given the impact of post-stroke spasticity, anti-spastic therapies have been developed as a continuous effort to manage the effects of spasticity on a multitude of levels, also including functional parameters (using the Trunk Impairment Scale, the Fugl-Meyer Motor and Sensory Assessments for the upper and lower limbs, Mini-Mental State Examination, Functional Ambulation Category, and Modified Barthel Index), pain intensity (using the Visual Analogue Scale, Numerical Rating Scales, the Verbal Rating Scales, and the Faces Pain Rating Scales), assessment of the time-course changes in neurologic impairments, and recovery in functional impairments (Activity of Daily Living, gait). Consequently, physical therapy, occupational therapy, self-rehabilitation, orthoses and assistive devices, pharmacological treatments, orthopaedic surgery, and neurosurgery are used for spasticity management [15,16,17,18,19]. Self-rehabilitation is a strategy that is added to conventional physical therapy to help patients maintain a satisfying level of physical activity after proper training by a physical or occupational therapist [20,21]. It has already been proven that programs of exercises that are provided by the therapists to be performed at home are highly appreciated by patients given the beneficial effects on functional capacity, motivation, and reliability, especially when the programs are associated with continuous professional support and guidance [21,22,23].

However, given the complexity of spasticity and previously reported methods lacking long-term efficacy and presenting adverse events, the management of this condition remains a demanding task, and it becomes essential to develop treatments and protocols that are focused on the symptoms as well as on the causes [14,15,24]. Anti-spastic medications may help to relieve the symptoms, but they may not provide functional benefit. Additionally, the efficacy of these medications may vary between individuals. During the rehabilitation process, treatments should address both the causes and symptoms and, therefore, the extensive comprehension of this phenomenon is essential and constant efforts are made in order to ensure an efficient way in which therapies act, providing a long-term, comprehensive management of spasticity.

Currently, excepting the utilization of botulinum toxin for the upper limb, there are no specific guidelines for the application of different non-pharmacological therapies in patients that are affected by spasticity [13,15]. Pharmacological treatments include oral medications, local injections, and intrathecal therapy. The most used oral medications consist of gamma aminobutyric acid agonists, central $\alpha_{2}$ receptor agonists, benzodiazepines, and muscle relaxants. Oral muscle relaxants reduce tone in both the spastic and non-spastic muscles, ending in generalized weakness and loss of function [25]. In addition, pharmacological tolerance frequently appears after few months of sustained use and the dose needs to be progressively increased in order to sustain the previous clinical effect and it may also induce adverse reactions [25,26]. Local injections consist of phenol or alcohol neurolysis and botulinum toxin. For example, it is known that long-term booster botulinum toxin injections may produce neutralizing antibodies that may alter the biological effects of this therapy [27,28,29,30]. Intrathecal therapy uses Baclofen and the risk of Baclofen overdose or withdrawal can be present [30]. Although evidence tends to be more accurate regarding the pharmacological treatment of spasticity, other non-pharmacological interventions for spasticity management lack a strong evidence base [31]. Additionally, the choice of treatment and goals may vary, depending on the extent and severity of the spasticity and on the functional status of every patient [24,30]. Physical therapy consists of different types of exercises (stretching exercises, range of motion exercises, weight bearing, balance oriented exercises, gait-training exercises, walking, task-specific functional training, aerobic training for fitness and endurance, fitting of splints or braces, etc.) and needs a daily program continuously adapted to every patient [13,30]. It is widely used in rehabilitation and it showed greater effectiveness in combination with other therapeutic interventions [30].

Extracorporeal shock wave therapy (ESWT) has been described as a potential therapeutic intervention in the management of post-stroke spasticity [32,33,34]. Shock waves are acoustic waves, which are omnipresent in our daily life: the sound of thunderstorms, an applauding crowd, and bangs of an explosion [35]. A shock wave is an acoustic pulse generating transient pressure disturbances with high peaks of pressure, short time life cycle, and rapid propagations in three-dimensional (3-D) space [36,37,38,39].

At the beginning, ESWT was first applied to disintegrate kidney and urethral stones [35,40]. Later on, shock waves have changed medical therapy in a substantial way by offering the possibility of a new approach for various ranges of existing conditions, such as tendinopathies and other musculoskeletal disorders [35,40,41,42,43]. This is still an underexplored field and more research is required in order to improve the understanding of such complex biological and medical effects of this therapy [35].

Recently, several reviews have evaluated the effects of ESWT on stroke survivors that are affected by spasticity, showing that ESWT is an effective intervention [13,14,44]. There are two types of ESWT, radial (rESWT) and focused (fESWT). In a randomized controlled trial [36], it was stated that applications of rESWT had positive effects on decreasing the level of spastic hypertonia of the upper limb muscles in patients with chronic stroke. However, there are still some methodological discrepancies to be considered; therefore, more consistent data need to be gathered and analyzed and the long-term effects should be monitored and quantified.

In a previous meta-analysis [45], ESWT demonstrated its efficacy on post-stroke spasticity. Nonetheless, it was not a meta-analysis only conducted on randomized controlled trials (RCTs), which might limit the quality of the studies and, therefore, the results. Furthermore, the effects of ESWT on post-stroke spasticity were mainly based on the Modified Ashworth Scale (MAS). Additionally, it did not take other factors that could have interfered with the results obtained into account: combined assessments and outcomes for both upper and lower limbs were included in the same meta-analysis; variation in number of patients (upper limb, *n* = 97 and lower limb, *n* = 63), which may have introduced bias in the analysis; only three of six studies continued follow-up at four weeks (two for upper limb and one for lower limb), which may minimize the reported effect of the therapy; the Modified Ashworth Scale was the only assessment method for spasticity grade; the baseline values varied across the studies; and, ESWT frequency and the number of sessions were not reported.

One systematic review and meta-analysis [44] did not present a follow-up period for all the included studies and predominantly used the MAS and Modified Tardieu Scale (MTS), lacking more accurate measures of spasticity assessment. Moreover, the study presented significant heterogeneity, which may have interfered with the results. Another systematic review and meta-analysis [13] used both RCTs and non-randomized controlled studies (NRS) and five of the included studies had an important risk of bias (PEDro score ≤3 points). The interpretation of the results might have been altered, given the poor quality of some studies. Other previous meta-analyses [46,47] revealed better reliability for measurements of upper limbs than for the lower limbs. In addition, [47] focused on both the upper and lower limbs, and also included different types of studies, showing variability within the parameters and results. Consequently, when considering the limitations of previous systematic reviews and meta-analyses, we performed a systematic review and meta-analysis of RCTs in order to investigate and assess the long-term effects of ESWT on lower limb post-stroke spasticity, as well as the adverse events encountered.

## 2. Materials and Methods

An evidence-based systematic review and meta-analysis was performed in accordance with the Preferred Reporting Items for Systematic Reviews and Meta-analyses (PRISMA) statement and it followed the recommendations of the Cochrane Handbook for Systematic Reviews [48,49]. The study’s protocol was published on PROSPERO International prospective register of systematic reviews website (study’s registration number: CRD42020207093).

### 2.1. Search Strategy and Eligibility Criteria

A systematic search of the electronic databases PubMed/MEDLINE, Physiotherapy Evidence Database (PEDro), Scopus, Ovid MEDLINE(R), and search engine of Google Scholar was conducted, while using the following key words: “extracorporeal shock wave therapy”, “stroke”, “spasticity”, and “lower limb”. Appendix A presents an example of the search strategy for PubMed/MEDLINE. Moreover, the authors checked the citation lists of all relevant trials for further references and additional relevant studies. Concerning the eligibility criteria, a selection was applied and publications ranging from January 2010 to August 2020 published in English, French, Spanish, Portuguese, and Italian language were included, and there were no country related restrictions. The concerned studies must have been conducted on humans, involve adults with lower limb post-stroke spasticity, and include both or any of the two types of ESWT (fESWT or rESWT). Patients who suffered an ischemic or hemorrhagic stroke, being in acute, subacute, or chronic phase, having a MAS score ≥1 were eligible. Only randomized controlled trials (RCTs) that were available as full-text were included. Non-randomized controlled trials, case reports, comments, papers, and letters were not taken into consideration. Duplicated data were also excluded.

### 2.2. Data Extraction

Study eligibility assessment and data extraction procedure were performed by two independent co-authors (E.E.M., L.D.). In the event of any disagreement, discussions and the opinion of a third author (M.B.) were used in order to reach consensus. Study selection was initially based on title and abstract and afterwards; full text articles were rigorously examined.

Two co-authors (E.E.M., L.D.) independently extracted the following relevant features of the included RCTs: the name of primary author and publication year, country, number of participants and mean age, interventions, including experimental group (EG) and control group (CG), time since stroke onset (mean), treated muscle, therapy site, tested muscle, outcome measures, side effects related to ESWT application, follow-up period and ESWT parameters: number of pulses, pressure, frequency, and duration of session.

### 2.3. Outcome Measures

The primary outcome was spasticity grade assessed mainly while using the MAS. Other scales, such as Modified Modified Ashworth Scale (MMAS) and MTS, were also used for spasticity grade assessment. Secondary outcomes were passive range of motion (PROM), pain intensity, gait assessment, electrophysiological parameters, and adverse events related to the ESWT application. The outcomes were classified in relation to the follow-up period after the ESWT intervention, as follows: short-term (immediately after treatment, thirty minutes after treatment, one hour after treatment, one week after treatment), and long-term (three weeks after treatment up to twelve weeks after treatment).

### 2.4. Quality Assessment

The risk of bias assessment of the included studies was independently performed by two co-authors (E.E.M., L.D.) while using the PEDro scale [50] and any disagreement was clarified by discussing it with a third author (M.B.). The risk of bias assessment includes the following domains: specified eligibility criteria, subjects randomly allocated to groups, allocation concealment, baseline comparability, blinding of all subjects, blinding of all therapists, blinding of all assessors, adequate measurement of outcome, intention to treat, between-group statistical comparisons, point measures, and measures of variability [50]. Using a funnel plot for publication bias assessment was not suitable, given the fact that only seven studies were selected.

### 2.5. Data Analysis

Standardized mean difference (SMD) with 95% confidence interval (95% CI) were used for summary of the statistical analysis. Sensitivity analysis was conducted when it was possible in order to evaluate the influence of a study on the overall effect. Heterogeneity of the selected studies was evaluated by $Chi^{2}$ test and $I^{2}$ value. $I^{2}$ values between 0% to 40%: the heterogeneity might not be important, 30% to 60%: may indicate moderate heterogeneity, 50% to 90%: may present substantial heterogeneity and, respectively, 75% to 100%: considerable heterogeneity is present [49]. A *p*-value < 0.05 was considered to be statistically significant. The statistical analyses were carried out while using Review Manager (RevMan) [Computer program]. Version 5.4. The Cochrane Collaboration, 2020, London, UK.

## 3. Results

**Figure 1:**
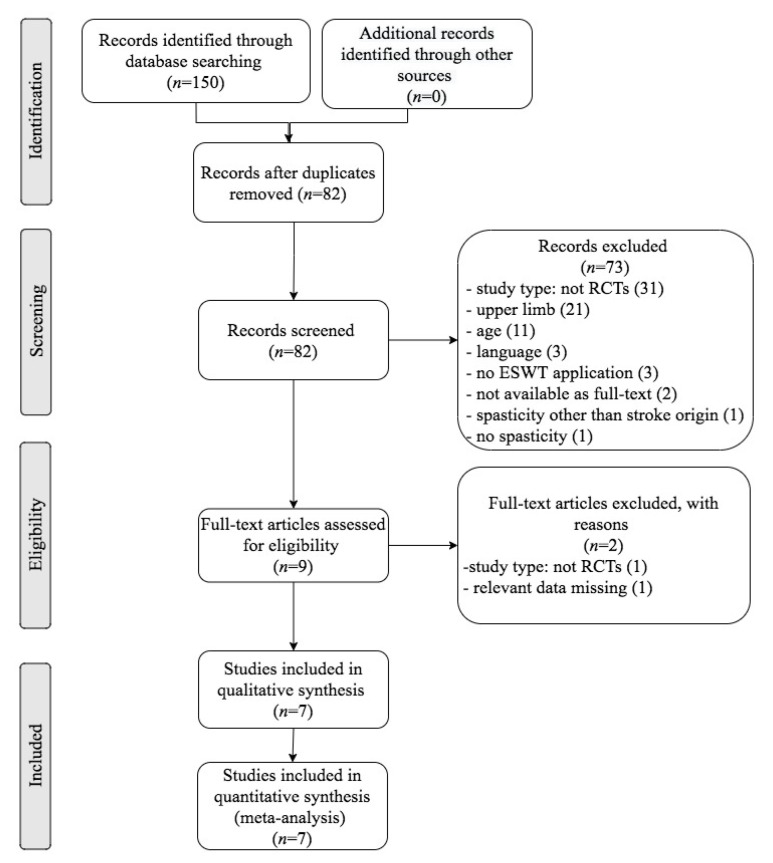
Flow diagram of the selection process of studies for the systematic review and meta-analysis according to The Preferred Reporting Items for Systematic Reviews and Meta-Analyses (PRISMA) guidelines.

Through a scientific literature search, 150 references were found and 82 remained after duplicates removal. After screening of title and abstract, 73 studies did not meet the eligibility criteria. After assessing the eligibility of the nine remaining full-text articles, two of them were excluded. Eventually, seven randomized controlled trials (RCTs) were included for the meta-analysis: Tirbisch, 2015; Taheri et al., 2017; Yoon et al., 2017; Wu et al., 2017; Radinmehr et al., 2017; Lee et al., 2019; Radinmehr et al., 2019 [32,33,51,52,53,54,55]. The Preferred Reporting Items for Systematic Reviews and Meta-Analyses (PRISMA) diagram (Figure 1) sums up the selection process and the results that were retrieved through literature search.

Table 1 presents an outline of the RCTs that were included with their associated characteristics and patient features. One author was contacted for clarifications, but, since no response was received, the study could not be included in the present systematic review and meta-analysis.

The seven selected randomized controlled trials included a total population of 170 subjects: 122 men and 48 women. When considering the stroke type, 54 participants were affected by ischemic stroke and 28 were affected by hemorrhagic stroke. Three RCTs did not report this information [33,53,54]. The seven RCTs include nine experimental groups and five control groups. In two studies, [32,54], no control group was used. The mean age of patients ranged from 44.11 to 66.9 years.

Lower limb spasticity was the main focus in the studies, although one of them evaluated the upper limb as well [32]. All of the RCTs included participants with MAS ≥1 and participants with fixed contractures were excluded. Regarding the type of ESWT used in the experimental groups, three studies used rESWT [33,51,54], two of them used fESWT [52,55], one study used both types of ESWT [32], and one study did not report the type of ESWT used [53]. As for the therapy site, two trials chose the myotendinous junction [51,52], one chose the muscle belly [32], one study chose both the muscle belly and myotendinous junction [53], two studies applied ESWT to the gastrocnemius bulk [33,54], and one study chose the medial head of the gastrocnemius muscle [55].

With regard to the anticoagulant medication, none of the studies presented data that were related to this type of medication. An important number of patients that were affected by stroke have indication of anticoagulants, as this medication plays a major role in the primary and secondary prevention of ischemic strokes. However, patients undergoing systemic anticoagulation therapy should consult their physicians regarding the temporary discontinuation of such medications before ESWT in order to prevent potential ecchymosis, hematoma, or bruising.

Concerning the adverse events that are related to ESWT intervention, one study described [51] mild pain during the first two sessions without hematoma or recrudescence of pain between sessions. In two other studies [32,54], patients reported no discomfort during the treatment and also presented no adverse events, such as skin petechiae, muscle hematoma, or focal edema.

The primary outcome was spasticity grade and its assessment was performed using different scales and measurements. Spasticity grade was assessed using the Modified Ashworth Scale, Modified Modified Ashworth Scale, and Modified Tardieu Scale. The secondary outcomes corresponded to a passive range of motion, pain intensity, functional mobility and balance, electrophysiological parameters, and adverse events.

For the clinical spasticity assessment, five studies used MAS [32,51,52,53,55] and two of them used Persian MMAS [33,54]. Only two trials used MTS [51,53]. With regard to the evaluation of the ESWT effect on post-stroke lower limb spasticity, the follow-up ranged from immediately after treatment up to twelve weeks.

The data were selected based on the experimental group and control group after ESWT and the effects were also evaluated before and after the ESWT intervention. All of the selected studies were randomized controlled trials and they stated the inclusion criteria.

The PEDro score assessing the risk of bias of the included trials ranged between 5 and 9, in this case allowing a stratification on two levels: high quality studies (=PEDro score 6–10) and fair quality studies (=PEDro score 4–5). The mean PEDro score was seven points out of 10 (Table 2).

Table 3 presents the ESWT parameters and duration of sessions. The parameters of ESWT intervention were quite similar in the trials. The number of pulses oscillated from 1500 to 2000 across the studies. Regarding the frequency, it ranged between 4 and 10 Hz, with 4 Hz and 5 Hz being the most used. Additionally, the pressure energy levels varied between 0.03 and 0.340 mJ/mm${}^{2}$. As for the duration of one ESWT session, only two studies reported this characteristic [51,54].

Multiple forest plots were performed while using the available analyzable data from the included studies, assessing the effects after ESWT application compared to the baseline. Additionally, control and experimental group comparisons were conducted.

**Figure 2:**
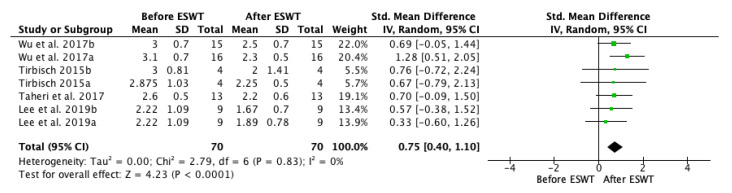
Forest plot of the standardized mean difference (SMD) and 95% confidence interval (95% CI) for spasticity assessed by Modified Ashworth Scale (MAS) before and after extracorporeal shock wave therapy (ESWT) on the short-term. Wu et al. 2017a: radial ESWT (rESWT); Wu et al. 2017b: focused ESWT (fESWT); Tirbisch 2015a: soleus muscle assessment; Tirbisch 2015b: gastrocnemius muscle assessment; Lee et al. 2019a: follow-up at 30 min. after ESWT; Lee et al. 2019b: follow-up at one week after ESWT.

A first forest plot (Figure 2) comparing the before and after ESWT effects on spasticity on the short-term was conducted based on the studies that presented analyzable data (including four studies and seven groups). A positive effect was found, favouring ESWT and showing efficacy on post-stroke spasticity on the short-term: standardized mean difference (SMD) = 0.75; 95% confidence interval (95% CI): (0.40–1.10); *p* < 0.0001.

**Figure 3:**
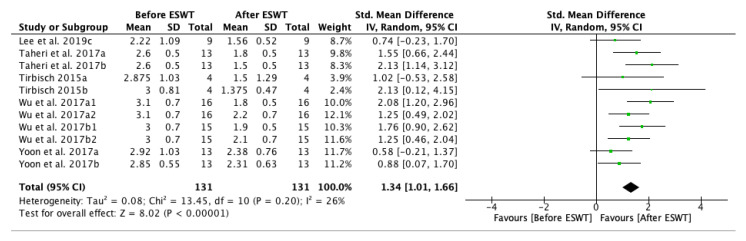
Forest plot of the standardized mean difference (SMD) and 95% confidence interval (95% CI) for spasticity assessed by Modified Ashworth Scale (MAS) before and after extracorporeal shock wave therapy (ESWT) on the long-term. Lee et al. 2019c: follow-up at four weeks after treatment; Taheri et al. 2017a: follow-up at three weeks after treatment; Taheri et al. 2017b: follow-up at 12 weeks after treatment; Tirbisch 2015a: soleus muscle assessment; Tirbisch 2015b: gastrocnemius muscle assessment; Wu et al. 2017a1: radial ESWT (rESWT) and follow-up at four weeks after treatment; Wu et al. 2017a2: radial ESWT (rESWT) and follow-up at eight weeks after treatment; Wu et al. 2017b1: focused ESWT (fESWT) and follow-up at four weeks after treatment; Wu et al. 2017b2: focused ESWT (fESWT) and follow-up at eight weeks after treatment; Yoon et al. 2017a: muscle belly application; Yoon et al. 2017b: myotendinous junction application.

The forest plot for spasticity assessment by MAS before and after extracorporeal shock wave therapy showed a positive effect on the long-term, up to twelve weeks after the treatment. Five of the studies were eligible for the meta-analysis. The MAS grade was significantly decreased after ESWT intervention: standardized mean difference (SMD) = 1.34; 95% confidence interval (95% CI): (1.01–1.66); and, *p* < 0.00001) (Figure 3).

**Figure 4:**
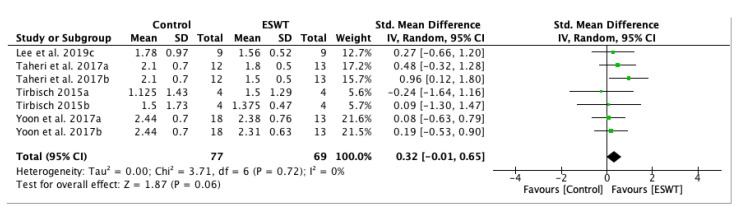
Forest plot of the standardized mean difference (SMD) and 95% confidence interval (95% CI) for spasticity assessed by Modified Ashworth Scale (MAS) comparing the control group (CG) and the experimental group (EG) after extracorporeal shock wave therapy (ESWT) on the long-term. Lee et al. 2019c: follow-up at four weeks after treatment; Taheri et al. 2017a: follow-up at three weeks after treatment; Taheri et al. 2017b: follow-up at 12 weeks after treatment; Tirbisch 2015a: soleus muscle assessment; Tirbisch 2015b: gastrocnemius muscle assessment; Yoon et al. 2017a: muscle belly application; Yoon et al. 2017b: myotendinous junction application.

A forest plot for spasticity assessment by MAS on the long-term comparing the control group and experimental group after ESWT application was performed. Four studies and seven groups presented analyzable data and they were included in the meta-analysis. The MAS score was decreased in the experimental group, favouring ESWT application: standardized mean difference (SMD) = 0.32; 95% confidence interval (95% CI): (−0.01–0.65); *p* = 0.06 (Figure 4).

**Figure 5:**
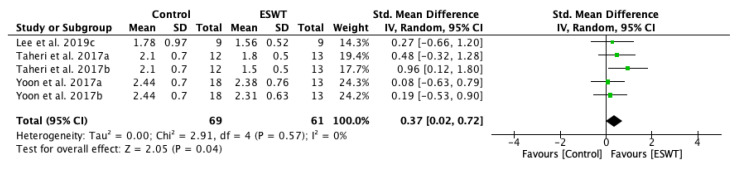
Forest plot of the standardized mean difference (SMD) and 95% confidence interval (95% CI) for spasticity assessed by Modified Ashworth Scale (MAS) comparing the control group (CG) and experimental group (EG) on the long-term. Sensitivity analysis. Lee et al. 2019c: follow-up at 4 weeks after treatment; Taheri et al. 2017a: follow-up at three weeks after treatment; Taheri et al. 2017b: follow-up at 12 weeks after treatment; Yoon et al. 2017a: muscle belly application; Yoon et al. 2017b: myotendinous junction application.

The results of the sensitivity analysis conducted on studies were consistent with the primary analysis, supporting the effects of ESWT intervention on decreasing spasticity on the long-term. The long-term effect of ESWT on decreasing spasticity was statistically significant: standardized mean difference (SMD) = 0.37; 95% confidence interval (95% CI): (0.02–0.72); *p* = 0.04 (Figure 5).

**Figure 6:**
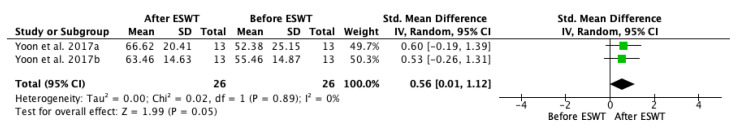
Forest plot of the standardized mean difference (SMD) and 95% confidence interval (95% CI) for spasticity assessed by Modified Tardieu Scale (MTS) before and after extracorporeal shock wave therapy (ESWT) on the long-term. Yoon et al. 2017a: muscle belly application; Yoon et al. 2017b: myotendinous junction application.

When assessing the ESWT effect on spasticity evaluated by Modified Tardieu Scale, a meta-analysis was performed and the results are presented in a forest plot (Figure 6). The MTS values were significantly improved in both the muscle belly group and the myotendinous junction group compared to the baseline: standardized mean difference (SMD) = 0.56; 95% confidence interval (95% CI): (0.01–1.12); *p* = 0.05. In both groups, the results tended to ameliorate after each ESWT session and the improvements progressed, whereas the therapy was continuing.

**Figure 7:**
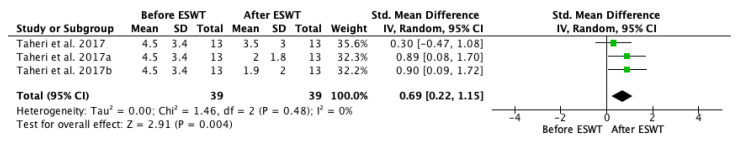
Forest plot of the standardized mean difference (SMD) and 95% confidence interval (95% CI) evaluating the short-term and long-term effects on pain intensity assessed by Visual Analogue Scale (VAS) before and after extracorporeal shock wave therapy (ESWT). Taheri et al. 2017: follow-up at one week after treatment; Taheri et al. 2017a: follow-up at three weeks after treatment; Taheri et al. 2017b: follow-up at 12 weeks after treatment.

The short-term and long-term effects of ESWT on pain score assessed by Visual Analogue Scale were presented in a new forest plot (Figure 7), showing that the pain intensity significantly decreased during the trial. It presented a significantly lower reduction at one week as compared to the baseline with a standardized mean difference (SMD) = 0.30; 95% confidence interval (95% CI): (−0.47–1.08). Moreover, the pain score continued to decrease between the first and third week after ESWT intervention. The effects at twelve weeks after treatment were similar to those at three weeks, showing a lasting effect of ESWT on reducing pain intensity. The standardized mean difference (SMD) = 0.69; 95% confidence interval (95% CI): (0.22–1.15); *p* = 0.004, being consistent with the results between the first week, the third week, and the twelfth week.

**Figure 8:**
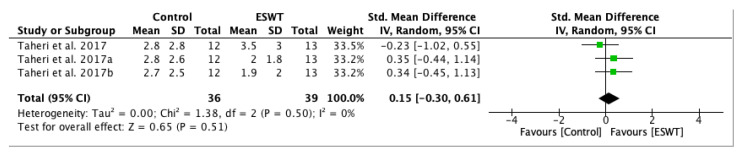
Forest plot of the standardized mean difference (SMD) and 95% confidence interval (95% CI) evaluating the short-term and long-term effects on pain intensity assessed by Visual Analogue Scale (VAS) comparing the control group (CG) and experimental group (EG) after extracorporeal shock wave therapy (ESWT). Taheri et al. 2017: follow-up at one week after treatment; Taheri et al. 2017a: follow-up at three weeks after treatment; Taheri et al. 2017b: follow-up at 12 weeks after treatment.

A meta-analysis was performed in order to assess short-term and long-term effects of ESWT on pain intensity on Visual Analogue Scale (Figure 8), showing that the pain intensity decreased more in the experimental group compared to the control group. However, it stated a significantly lower reduction in the experimental group at one week when compared to the control group with a standardized mean difference (SMD) = −0.23; 95% confidence interval (95% CI): (−1.02–0.55). The pain score continued to decrease between the first and third week after ESWT intervention in both groups, but it presented a statistically significant reduction in the experimental group. The lasting effects were seen at twelve weeks after treatment and the findings are also consistent with the before and after ESWT analysis. The standardized mean difference (SMD) = 0.15; 95% confidence interval (95% CI): (−0.30–0.61); *p* = 0.51.

**Figure 9:**
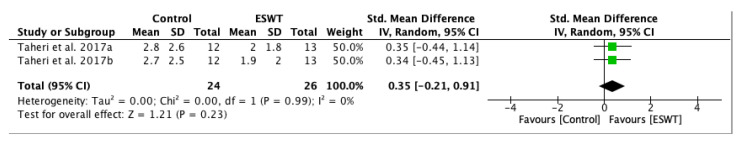
Forest plot of the standardized mean difference (SMD) and 95% confidence interval (95% CI) evaluating long-term effects on pain intensity assessed by Visual Analogue Scale (VAS) comparing the control group (CG) and the experimental group (EG) after extracorporeal shock wave therapy (ESWT). Taheri et al. 2017a: follow-up at three weeks after treatment; Taheri et al. 2017b: follow-up at 12 weeks after treatment.

A forest plot was conducted in order to evaluate long-term effects of ESWT on pain score assessed by Visual Analogue Scale (Figure 9), favouring ESWT intervention in the long-term. However, between the third week and the twelfth week there is not a significant difference, but the overall effect of ESWT intervention lasted. The standardized mean difference (SMD) = 0.35; 95% confidence interval (95% CI): (−0.21–0.91); *p* = 0.23.

**Figure 10:**
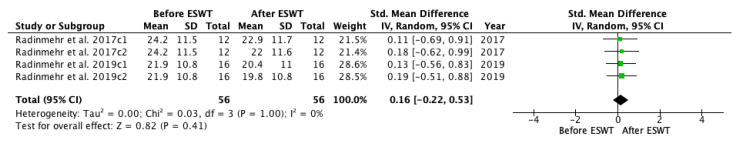
Forest plot of the standardized mean difference (SMD) and 95% confidence interval (95% CI) evaluating the short-term effects for Timed Up and Go Test (TUG) before and after extracorporeal shock wave therapy (ESWT). Radinmehr et al. 2017c1: follow-up immediately after treatment; Radinmehr et al. 2017c2: follow-up at 1 h after the end of treatment; Radinmehr et al. 2019c1: follow-up immediately after treatment; Radinmehr et al. 2019c2: follow-up at 1 h after the end of treatment.

For the evaluation of the functional mobility and balance, the Timed Up and Go Test (TUG) was used. Only two studies used this outcome and the analyzable data allowed for conducting a meta-analysis (Figure 10), showing a positive effect of ESWT on this parameter. After one ESWT session, an improvement was observed immediately and one hour after the treatment, showing that, even after the end of therapy, the effect lasted. There was a progressive reduction in TUG test scores, although there was not a significant difference between the results that were obtained immediately and one hour after the treatment.

**Figure 11:**
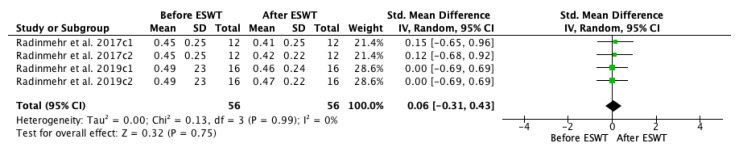
Forest plot of the standardized mean difference (SMD) and 95% confidence interval (95% CI) evaluating the short-term effects on $H_{max}$/$M_{max}$ ratio before and after extracorporeal shock wave therapy (ESWT). Radinmehr et al. 2017c1: follow-up immediately after treatment; Radinmehr et al. 2017c2: follow-up at 1 h after the end of treatment; Radinmehr et al. 2019c1: follow-up immediately after treatment; Radinmehr et al. 2019c2: follow-up at 1 h after the end of treatment.

A meta-analysis on $H_{max}$/$M_{max}$ ratio before and after ESWT intervention was conducted (Figure 11), showing that significant improvements were not found across assessments immediately and one hour after the treatment as compared to the baseline. The standardized mean difference (SMD) = 0.06; 95% confidence interval (95% CI): (−0.31–0.43); *p* = 0.75.

**Figure 12:**
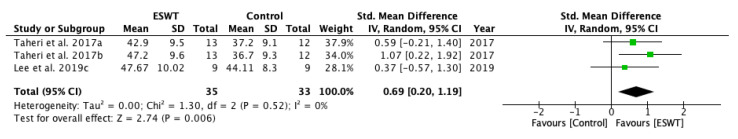
Forest plot of the standardized mean difference (SMD) and 95% confidence interval (95% CI) evaluating the long-term effects on passive range of motion (PROM) before and after extracorporeal shock wave therapy (ESWT). Taheri et al. 2017a: follow-up at 3 weeks after treatment; Taheri et al. 2017b: follow-up at 12 weeks after treatment; Lee et al. 2019c: follow-up at four weeks after treatment.

For the assessment of long-term effects of ESWT intervention on PROM, two studies, including three groups, were selected for meta-analysis (Figure 12). A statistically significant effect favouring ESWT was found when comparing the control and the experimental group, showing that the effects lasted up to twelve weeks after the treatment. The standardized mean difference (SMD) = 0.69; 95% confidence interval (95% CI): [0.20–1.19]; *p* = 0.006.

**Figure 13:**
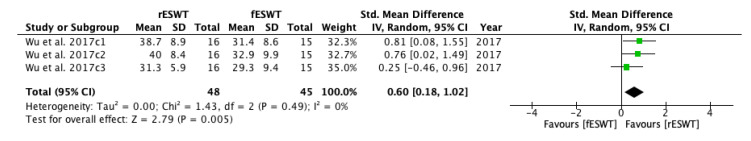
Forest plot of the standardized mean difference (SMD) and 95% confidence interval (95% CI) evaluating short-term and long-term effects on passive range of motion (PROM) comparing focused extracorporeal shock wave therapy (fESWT) to radial extracorporeal shock wave therapy (rESWT). Wu et al. 2017c1: follow-up at 1 week after radial ESWT (rESWT) and focused ESWT (fESWT); Wu et al. 2017c2: follow-up at 4 weeks after radial ESWT (rESWT) and focused ESWT (fESWT); Wu et al. 2017c3: follow-up at 8 weeks after radial ESWT (rESWT) and focused ESWT (fESWT).

A meta-analysis was conducted for the overall effects of fESWT and rESWT (Figure 13). Through selection, one study, including three groups with analyzable data, stated a statistically significant effect of rESWT on PROM: the standardized mean difference (SMD) = 0.60; 95% CI confidence interval (95% CI): (0.18–1.02); *p* = 0.005. These results are consistent with the effects on spasticity assessed by MAS and MTS and with the pain intensity that is evaluated by VAS.

No significant heterogeneity was found across the studies and groups that were included in the conducted meta-analyses.

## 4. Discussion

ESWT intervention on post-stroke spasticity became more intensively used during the last years, proving its effectiveness [56]. Nonetheless, long-term efficacy, especially for the lower limb, is still not entirely evaluated. Therefore, the aim of this systematic review and meta-analysis was to assess long-term effects of ESWT used as an innovative intervention on post-stroke lower limb spasticity in adults. Adverse events were also taken into account because the safety profile is an utterly important part of any therapy. According to the study design and protocol, the seven included trials were represented by RCTs (six of them focused only on lower limb spasticity and one study [32] evaluated both the upper limb and the lower limb spasticity). The present systematic review and meta-analysis focused on RCTs, being known as studies with the highest reliability and quality of research data [57].

A selection of different outcome measures and various follow-up evaluations were conducted and evaluated across the selected studies. By analyzing them on both the short-term and long-term, the goal was to give insight into the variations of ESWT efficacy during time.

With regard to spasticity assessment, the meta-analysis demonstrated a statistically significant effect of ESWT on reducing the lower limb post-stroke spasticity on the short-term as well as on the long-term. The results are consistent with findings of a previous systematic review and meta-analysis [13], but different to those that are presented in other systematic reviews and meta-analyses [44,45], evaluating both the upper limb and lower limb, which might have influenced the variables, leading to differences between the results.

The analyzed studies in terms of PROM revealed a statistically significant improvement on the long-term in the experimental group as compared to the control group after ESWT.

Regarding pain intensity that is assessed by VAS, ESWT intervention indicated a statistically significant reduction both on short-term and long-term, proving a lasting efficacy of ESWT on this parameter. However, the initial VAS score for the experimental group showed a higher intensity of pain when compared to the control group at baseline, which may underestimate the ESWT effect on decreasing pain intensity. There is an impetuous need for high quality studies to be conducted and follow-up the long-term effects on this parameter to improve the patient health-related quality of life, as only one study evaluated this parameter and provided analyzable data [52].

The ESWT intervention on gait evaluated by TUG did not show a significant difference. Because only one study had a control group [33], only before and after ESWT comparisons could have been performed. The statistical significance for TUG could not be assessed, since it is customary to determine a size that is greater than 23% of the clinical change to be considered as a clinically meaningful improvement [33,54,58]. The obtained results could be explained by the fact that the assessment was held immediately and one hour after the treatment, and only one ESWT session was performed. Moreover, sessions of physical therapy were not held within the groups. However, given the gait pattern and its complexity, more sessions may be needed and the assessment should also be held on the long-term [33,54,58].

As a tool for spasticity assessment, the MAS was used the most and it showed better effectiveness than the electrophysiological parameters. Concerning spasticity, the MAS is widely used in the clinical assessment because of the ease in operating with it [59]. However, it relies on subjective evaluation and it also does not entirely adhere to the velocity dependent definition of spasticity [7,59,60]. MTS is more related to velocity dependence of the spasticity definition and, when comparing MAS and MTS in spasticity measurements in children with cerebral palsy, MTS seemed to prove a better reliability as a method of measurement [61,62]. Although MAS is the most frequently used scale to assess spasticity, the high variation in reliability may be the result of the deficiency of standardization [63].

So far, the mechanisms underlying the effects of ESWT intervention on spasticity have not been fully explored. In the scientific literature, three hypotheses are formulated for describing the complex biochemical mechanisms that are related to ESWT activity: the production of nitric oxide (NO), the effect on spinal cord excitability, and the action on the Golgi tendon organ (GTO) [34,64,65,66,67,68,69,70,71]. ESWT is believed to induce NO production, which is involved in neurotransmission, memory formation, and synaptic plasticity in the central nervous system (CNS), also taking part in the activity of the neuromuscular junctions of the peripheral nervous system [34,64]. As the main outcome was spasticity, NO was considered to be a possible factor in reducing it by improving muscle stiffness [34,65,72]. Specifically, ESWT intervention on the muscles and tendons was found to produce a long-term tissue regeneration effect in addition to having an immediate anthalgic and anti-inflammatory outcome [64]. Moreover, an increase in neoangiogenesis in the tendons of dogs was observed after four to eight weeks of ESWT application [64]. Although the mechanisms underlying the anti-inflammatory effects of ESWT are not fully elucidated, the data indicate the significant role that is played by NO in the therapeutic effect.

With regard to the decrease of the excitability of alpha motor neuron, accurate data are still insufficient. According to previously undertaken studies, patients with ameliorated MAS score did not necessarily show a better response in $H_{max}$/$M_{max}$ ratio, which suggests that ESWT may not have an influence on the improvement of alpha motor neuron excitability [34,69]. In terms of $H_{max}$/$M_{max}$ ratio after ESWT intervention, no significant improvements were found across assessments in two of the included studies [33,54]. These results are consistent with previous studies on lower limb post-stroke spasticity [67,69].

Because, after ESWT intervention, no significant changes in F-wave latency and H-reflex latency were observed, the effect on excitability of the spinal cord and Golgi tendon could be excluded as the leading mechanism. These findings are consistent with the results from different studies [34,69]. However, the electrophysiological parameters are still to be explored and they should be further analyzed through high quality studies along with ultrasonographic parameters in order to provide more accurate insight into modifications on spasticity of stroke etiology.

The number of ESWT sessions is an important feature for treatment effectiveness. In this systematic review and meta-analysis, the number of sessions varied between one session in three of the studies [33,54,55], three sessions in another three trials [32,52,53], and nine sessions in one study [51]. Important variability is present, even though the shock wave parameters, such as pressure and frequency, are similar. Larger high quality studies should be undertaken and protocols could be developed in order to ensure a satisfying safety profile and assess long-lasting effectiveness through this non-invasive type of therapy.

The relatively young age of the participants in the studies included in our systematic review and meta-analysis (the mean age of patients ranged from 44.11 to 66.9 years) could be explained by the increasing incidence of stroke in young people. Stroke at young age is an increasing health related problem in both developing and developed countries due to the rising incidence and high morbidity and mortality. Stroke in young adults stands more heterogeneity when compared to stroke in older adults due to the multitude of possible risk factors and etiologies. Differences in geographical regions, ethnicity, and sex, as well as exposure of vascular risk factors could partially explain the incidence variation of ischemic stroke in young adults that has been observed all over the world [73].

Concerning the adverse events that are related to ESWT intervention, no reactions at all or only mild adverse reactions were described. Given the novelty of the technique for spasticity treatment, it is of great importance to keep investigating whether any immediate or long-term adverse events occur, trying to avoid or limit them to maintain a satisfactory safety profile of the ESWT intervention.

Admittedly, the current study also has several limitations. Firstly, there is a limited number of RCTs available, which restricted the number of studies that were included in the present systematic review and meta-analysis. Additionally, some of the studies had a small number of participants. Nonetheless, the current systematic review and meta-analysis only included those randomized controlled trials with at least a satisfying PEDro score. Secondly, findings on adverse events were not available in all of the selected studies. However, the reported adverse effects were mild, manageable and most of them solved in a few days time. Thirdly, not all trials provided analyzable data on all outcome measures and they did not provide long-term follow-up evaluations in order to compare the effects during time. Fourthly, not all of the trials presented a control group and, among those presented, there is a lack of consistency with respect to the intervention. Finally, variability was found across the outcomes and assessments.

Further high-quality research studies with a greater number of participants are needed in order to clarify more particular aspects about ESWT, including more objective outcome measures, shock wave delivery, intensity, duration, safety profile, and protocols.

## 5. Conclusions

The present systematic review and meta-analysis concludes that ESWT ameliorated lower limb spasticity in stroke survivors and its efficacy lasted up to twelve weeks, proving its long-term effectiveness. In addition, ESWT also reduced pain intensity and increased the range of motion. The therapeutic intervention did not present significant short-term and long-term adverse events, proving a satisfactory safety profile. These results need to be further confirmed and future research should focus on protocols, the duration of the intervention, parameters, objective outcome measures, and safety profile.

## Figures and Tables

**Table 1:** Characteristics of the randomized controlled trials included in the systematic review and meta-analysis.

Study	Country	Participants n (M/F)	Mean Age (Year)	Type of Stroke (Ischemic/Hemorrhagic)	Interventions	Time Since Onset Mean ± SD (Months)	Treated Muscle	Therapy Site	Sessions	Tested Muscles	Outcome Measures	Side Effects	Follow-Up
Tirbisch [51]	France	8 (3/5)	49.5 ± 8.736 61.25 ± 11.116	3/1 3/1	EG: rESWT+CP CG: CP	3.97 ± 0.83 3.43 ± 1.63	gastrocnemius muscle	myotendinous junction	9	soleus muscle gastrocnemius muscle	MAS, MTS	Pain	$T_{0}$ (initial) $T_{1}$ (after the 1st session) $T_{2}$ (at the end of the 9th session, week 3)
Taheri et al. [52]	Iran	25 (17/8)	56.5 ± 11.6 54.9 ± 9.4	11/2 11/1	EG: fESWT+anti-spastic medications + stretching exercises CG: Anti-spastic medications + stretching exercises	33 ± 21.4 25.8 ± 9.9	gastrocnemius muscle	myotendinous junction	3	ankle plantar flexor	MAS, PROM, VAS Clonus score, 3-MWD, LEFS	NA	$T_{0}$ (baseline) $T_{1}$ (one week after treatment) $T_{2}$ (3 weeks after treatment) $T_{3}$ (12 weeks after treatment)
Yoon et al. [53]	Korea	44 (42/2)	61.0 ± 12.2 66.9 ± 4.9 59.5 ± 16.9	NR	EG: Active ESWT muscle belly group active ESWT myotendinous junction group CG: sham ESWT, only sound over the muscle without any transducer contact	99.1 ± 85.1 51.1 ± 36.0 38.7 ± 30.2	semitendinosus muscle	muscle belly myotendinous junction	3	knee flexor	MAS, MTS	NA	$T_{0}$ (baseline) $T_{1}$ (1 week) $T_{2}$ (2 weeks) $T_{3}$ (3 weeks)
Wu et al. [32]	Taiwan	31 (18/13)	60.3 ± 9.9 59.6 ± 11.3	10/5 10/6	EG: fESWT rESWT CG: NA	53.2 ± 26.7 55.7 ± 26.1	triceps surae muscle	muscle belly	3	gastrocnemius muscle	MAS, MTS, PROM, dynamic foot contact area, gait speed, AE	None	$T_{0}$ (prior to treatment) $T_{1}$ (one week after treatment) $T_{2}$ (4 weeks after treatment) $T_{3}$ (8 weeks after treatment)
Radinmehr et al. [54]	Iran	12 (7/5)	59.0 ± 13	NR	EG: rESWT CG: NA	34.3 ± 20.6	plantar flexor muscle	gastrocnemius bulk	1	gastrocnemius muscle soleus muscle	$H_{max}/M_{max}$ ratio, H-reflex latency, Persian MMAS, AROM, PROM, PPFT, TUG Test	None	$T_{0}$ (baseline) $T_{1}$ (immediately after treatment) $T_{2}$ (one hour after the end of the treatment)
Lee et al. [55]	Korea	18 (16/2)	50.89 ± 8.81 44.11 ± 4.07	4/5 2/7	EG: fESWT CG: sham stimulation	12.89 ± 8.99 10.44 ± 9.11	gastrocnemius muscle	medial head of the gastrocnemius muscle	1	gastrocnemius muscle	MAS, PROM, FMA, US measures: ATL, MFL, MT, PA	NA	$T_{0}$ (prior to treatment) $T_{1}$ (30 min after treatment) $T_{2}$ (1 week after treatment) $T_{3}$ (4 weeks after treatment)
Radinmehr et al. [33]	Iran	32 (19/13)	56.0 ± 12.3 56.2 ± 8.4	NR	EG: rESWT US group: continuous ultrasound	34.4 ± 20.5 36.8 ± 15.1	gastrocnemius muscle	gastrocnemius bulk	1	knee flexors and knee extensors	Persian MMAS, AROM, PROM, PPFT, TUG Test, H-reflex test, $H_{max}/M_{max}$ ratio	NA	$T_{0}$ (baseline) $T_{1}$ (immediately after treatment) $T_{2}$ (1 h after the end of the treatment)

Abbreviations: AE, adverse events; AROM, active range of motion; ATL, Achilles tendon length; CG, control group; CP, conventional physiotherapy; EG, experimental group; ESWT, extracorporeal shock wave therapy; fESWT, focused shock wave therapy; FMA, Fugl-Meyer Assessment; H-reflex, Hoffmann Reflex; $H_{max}/M_{max}$ ratio, ratio of maximum Hoffmann Reflex to maximum M response; LEFS, Lower Extremity Functional Score; MAS, Modified Ashworth Scale; MFL, muscle fascicle length; M/F, Male/Female ratio; MMAS, Modified Modified Ashworth Scale; MT, muscle thickness; MTS, Modified Tardieu Scale; NA, not available; NR, not reported; PA, Pennation Angle; PPFT, passive plantar flexor torque; PROM, passive range of motion; rESWT, radial shock wave therapy; TUG, Timed Up and Go test; US, ultrasound; US measures, ultrasonographic measures; VAS, Visual-Analogue Scale; 3-MWD, 3-meter Walk Duration.

**Table 2:** Risk of bias assessment of selected randomized controlled trials by PEDro scale.

Items	Tirbisch (2015)	Taheri et al. (2017)	Yoon et al. (2017)	Wu et al. (2017)	Radinmehr et al. (2017)	Lee et al. (2019)	Radinmehr et al. (2019)
Eligibility criteria were specified ^1^	yes	yes	yes	yes	yes	yes	yes
Subjects were randomly allocated to groups	yes	yes	yes	yes	yes	yes	yes
Allocation concealment	yes	yes	no	yes	no	yes	no
Baseline comparability	yes	yes	yes	yes	yes	yes	yes
Blinding of all subjects	no	no	no	yes	no	yes	no
Blinding of all therapists	no	no	no	no	no	yes	no
Blinding of all assessors	yes	no	no	yes	yes	yes	yes
Adequate outcome measures and follow-up	yes	yes	yes	yes	yes	yes	yes
Intention to treat analysis	yes	no	no	no	no	no	yes
Between-group statistical comparisons	yes	yes	yes	yes	yes	yes	yes
Point estimates and variability measures	yes	yes	yes	yes	yes	yes	yes
**PEDro score**	**8**	**6**	**5**	**8**	**6**	**9**	**7**

^1^ This criterion influences external validity, but not the internal or statistical validity of the trial. This item does not contribute to the calculation of the PEDro score. Abbreviations: PEDro, Physiotherapy Evidence Database.

**Table 3:** Selected studies and extracorporeal shock wave therapy parameters.

Study	Number of Pulses/Shots	Energy/Pressure	Frequency (Hz)	Duration of Session
Tirbisch [51]	2000	0.03 mJ/mm${}^{2}$; 2.5 bar	10 Hz	15 min
Taheri et al. [52]	1500	0.1 mJ/mm${}^{2}$	4 Hz	NA
Yoon et al. [53]	1500	0.068–0.093 mJ/mm${}^{2}$	5 Hz	NA
Wu et al. [32]	1500	0.10 mJ/mm${}^{2}$; 2 bar	5 Hz	NA
Radinmehr et al. [54]	2000	0.34 mJ/mm${}^{2}$; 60 mJ (1 bar)	5 Hz	7 min
Lee et al. [55]	2000	0.1 mJ/mm${}^{2}$	4 Hz	NA
Radinmehr et al. [33]	2000	0.34 mJ/mm${}^{2}$; 60 mJ (1 bar)	5 Hz	NA

Abbreviations: NA, not available.

## Appendix A. Search Strategy for PubMed/MEDLINE

1. stroke*[tiab] OR poststroke*[tiab] OR hemiparesis OR hemiplegia OR cerebrovascular disorders [Mesh] OR infarction OR “brain vascular accidents”

2. shockwaves [MeSH Terms] OR shock [All Fields] waves [All Fields] OR shock wave therapy [MeSH Terms] OR shock wave therapy [All Fields] AND stroke [MeSH Terms] OR stroke [All Fields] OR poststroke [MeSH Terms] OR poststroke [All Fields] AND spasticity [MeSH Terms] OR spasticity [All Fields] OR hypertonicity [MeSH Terms] OR hypertonicity [All Fields] OR increased tone [MeSH Terms] OR increased tone [All fields] AND lower limb [MeSH Terms] OR lower limb [All Fields] OR lower extremity [MeSH Terms] OR lower extremity [All fields]

3. shockwaves [MeSH Terms] OR shock [All Fields] waves [All Fields] OR shock wave therapy [MeSH Terms] AND stroke [MeSH Terms] OR [All Fields] OR poststroke [MeSH Terms] AND spasticity [MeSH Terms] OR spasticity [All Fields] OR hypertonicity OR increased tone [MeSH Terms] AND lower limb

4. (stroke*[tiab] OR poststroke*[tiab] OR hemiparesis OR hemiplegia OR cerebrovascular disorders [Mesh] OR infarction OR “brain vascular accidents”) AND (spasticity [tiab] OR muscle hypertonia [tiab] OR “muscular hypertonicity” OR “muscular hypertonia” OR “increased tone” OR exaggerated)

5. (shockwave OR shock waves therapy OR “extracorporeal shock waves” OR ESWT) AND (stroke*[tiab] OR poststroke*[tiab] OR hemiparesis OR hemiplegia OR cerebrovascular disorders [Mesh] OR infarction) AND ((spasticity [tiab] OR muscle hypertonia [tiab] OR “muscular hypertonicity” OR “muscle hypertonia” OR “increased tone”)
